# Conformity to traditional Mediterranean diet and cancer incidence: the Greek EPIC cohort

**DOI:** 10.1038/sj.bjc.6604418

**Published:** 2008-07-01

**Authors:** V Benetou, A Trichopoulou, P Orfanos, A Naska, P Lagiou, P Boffetta, D Trichopoulos

**Affiliations:** 1 Department of Hygiene, Epidemiology and Medical Statistics, University of Athens Medical School, 75 Mikras Asias street, Athens 115 27, Greece; 2 Genetics and Epidemiology Cluster, International Agency for Research on Cancer (IARC), 150 Cours Albert-Thomas, Lyon 69008, France; 3 Department of Epidemiology, Harvard School of Public Health, 677 Huntington Avenue, Boston, MA 02115, USA; 4 Hellenic Health Foundation, 10–12 Tetrapoleos street, Athens 115 27, Greece

**Keywords:** Mediterranean diet, cancer incidence, dietary patterns, Greece, EPIC Study, cohort study

## Abstract

Adherence to traditional Mediterranean diet (MD) has been reported to be inversely associated with total, as well as cardiovascular, mortality. We have examined the relation between degree of such adherence and incidence of cancer overall in a general population sample of 25 623 participants (10 582 men, 15 041 women) of the Greek segment of the European Prospective Investigation into Cancer and nutrition (EPIC). All subjects completed a validated, interviewer-administered, semi-quantitative food-frequency questionnaire at enrolment. Degree of adherence to the traditional MD was assessed through a 10-point scale (0 minimal; 9 maximal) that incorporated key dietary characteristics. During a median follow-up of 7.9 years and 188 042 total person-years, 851 medically confirmed incident cancer cases (421 men, 430 women) were recorded. Using proportional hazards regression with adjustment for potential confounders, we found that a higher degree of MD adherence was associated with lower overall cancer incidence. A two-point increase in the score corresponded to a 12% reduction in cancer incidence (adjusted hazard ratio, 0.88 (95% confidence interval 0.80, 0.95)). The association was exposure-dependent and stronger among women. This inverse association with MD adherence was considerably stronger than that predicted on the basis of the associations of the individual components of this diet and points to the value of analysing dietary patterns in cancer studies.

Traditional Mediterranean diet (MD) prevailed in the olive oil growing areas of the Mediterranean region up to the early 1960s; olive oil which is its central element facilitates the consumption of large quantities of vegetables and legumes (Trichopoulou and Lagiou, 1997). Adherence to the traditional MD has been studied using a 9-point scale (1) high monounsaturated to saturated lipid ratio, (2) high consumption of fruits, (3) high consumption of vegetables, (4) high consumption of legumes, (5) high consumption of cereals, (6) moderate-to-high consumption of fish, (7) low consumption of meat and meat products, (8) low-to-moderate consumption of milk and dairy products and (9) moderate consumption of ethanol, mostly in the form of wine at meals (Trichopoulou *et al*, 1995, 2003). Several other scales assessing conformity to MD also exist (Fung *et al*, 2005; Trichopoulou *et al*, 2005a, Bach *et al*, 2006).

MD has received much attention as a diet associated with lower risk of coronary heart disease (Keys, 1980). Several reports, however, have indicated that this may extend to several forms of cancer (Trichopoulou *et al*, 2000b, 2003; Bosetti *et al*, 2003; Lagiou *et al*, 2006; Mitrou *et al*, 2007). We have examined the degree of adherence to traditional MD in relation to incidence of cancer overall in a prospective cohort investigation in Greece. Two advantages are relevant here: the cohort is based on the general population and large segments of it still adhere to the traditional MD (The Data Food Networking, 2008).

## Materials and methods

A total of 28 572 participants from all over Greece were recruited in 1994–99 in the Greek component of European Prospective Investigation into Cancer and nutrition (EPIC), the prospective cohort study investigating the role of biologic, dietary, lifestyle and environmental factors in the aetiology of cancer and other chronic diseases. It is conducted in 23 research centres in 10 European countries (Riboli *et al*, 2002). For Greece, the study protocol was approved by the ethics committees of the International Agency for Research on Cancer (IARC) and the University of Athens Medical School. All participants provided written informed consent and all procedures were in accordance with the Helsinki Declaration.

A validated, semi-quantitative, food-frequency questionnaire including 150 foods and beverages, as well as several complex recipes, was used to assess usual intake during the year preceding enrolment (Gnardellis *et al*, 1995; Katsouyanni *et al*, 1997). The questionnaire was administered in person by trained interviewers. For each item, participants were asked to report their frequency of consumption and portion size with the help of 76 photographs of portion sizes from which quantities consumed in grams per day were estimated. Nutrient and total energy intakes were calculated for each participant using a food-composition database modified to accommodate the particularities of the Greek diet (Trichopoulou and Georga, 2004). For this analysis, 11 all-inclusive foods groups as well as selected nutrients were considered: vegetables, legumes, fruits and nuts, dairy products, cereals, meat and meat products, fish and shellfish, potatoes, eggs, confectionery and non-alcoholic beverages, as well as ethanol, monounsaturated (mostly from olive oil), polyunsaturated and saturated lipids.

A 10-point scale was used to assess the degree of adherence to the traditional MD (Trichopoulou *et al*, 2003). A value of 0 or 1 was assigned for each of the nine components indicated below, using the sex-specific median as the cutoff value. For the presumed beneficial components (vegetables, legumes, fruits and nuts, cereals, and fish), participants were assigned a value of 0 for consumption below the median and of 1 for consumption at or above the median. For the presumed detrimental components (meat and meat products, and dairy products) the value of 0 was assigned for consumption at or above the median, while a value of 1 was assigned for below the median. For ethanol a value of 1 was assigned to men consuming 10 g to less than 50 g per day and to women consuming 5 g to less than 25 g per day. For lipid intake, a value of 1 was assigned to individuals with a monounsaturated to saturated lipids ratio at or above the median. Thus, the final MD score ranged from 0 (minimal adherence) to 9 (maximal).

A number of socio-demographic and lifestyle characteristics, including smoking, physical activity and use of dietary supplements were recorded at enrolment. Anthropometric measurements were also undertaken using standardised procedures and allowing for the calculation of body mass index (in kg/m^2^). Finally, an overall metabolic equivalent (MET)-hour per day index, expressing the average daily energy expenditure level, was calculated from the frequency and duration of participation in occupational and leisure-time physical activities (Ainsworth *et al*, 1993; Trichopoulou *et al*, 2000a. 2003).

From the original cohort of 28 572 participants, 1696 (5.9%) were excluded because follow-up information was not available, 383 participants (1.3%) due to prevalent cancer at enrolment (excluding non-melanoma skin cancer) and another 870 (3.0%) due to missing values in one or more analysed variables. The final study population consisted of 25 623 individuals.

Active follow-up methods were implemented by specially trained health professionals through telephone interviews with the participants or, in case of death, their next of kin. A structured form was completed for each incident cancer and the diagnosis was verified through pathology reports, medical records, discharge diagnoses or death certificates, according to IARC guidelines for end point data in EPIC. Cancers were classified according to the International Classification of Diseases for Oncology (ICD-O-2) (World Health Organization, 1990). The outcome studied was the first cancer diagnosis (excluding non-melanoma skin cancer), or death from cancer whenever diagnosis was not reported.

Frequency distributions were used for descriptive purposes. Means and s.d. were used for dietary variables, including energy intake. Cox proportional hazards regression, with time to event as the primary time variable, was used to assess relationships with, alternatively, each dietary variables (per increment of intake calculated as a round number close to the s.d. of the relevant daily intake) or the MD score. The date of first diagnosis of cancer (or of death from incident cancer when diagnosis date was not reported) was used to calculate time to event. All models were adjusted for age, years of schooling, smoking status, body mass index, height, physical activity expressed in MET-hours per day, ethanol intake, supplement intake and total energy intake. In analyses of MD score and cancer, consumption of potatoes, eggs, confectionery and nonalcoholic beverages (which are not components of the score) were also controlled for (continuously), whereas ethanol (which is a component of the score) was not. Stratification by sex was also performed (except for the sex-specific models). In the Cox models, the assumption of proportionality (assessed through the Schoenfeld goodness of fit test) was met, no time-dependent covariates were used and there was no colinearity among the variables. All analyses were performed using STATA 8.0 statistical package (Stata Corporation, 2003).

## Results

During the follow-up period of 25 623 participants to March 2007 (median of 7.9 years, with a total of 188 042 person-years), 851 incident cancers (excluding nonmelanoma skin cancer) were diagnosed (of which 110 cases died from cancer without an earlier date of diagnosis). Table 1 shows baseline characteristics of participants and the cancers by sex and MD score. The table indicates that those with higher MD score tend to be younger, more educated and physically more active. These variables, however, as well as the others shown in Table 1 were controlled for in the multivariate analysis.

Table 2 shows the distribution of cancers by site and sex. Among men, lung cancer is commonest, followed by cancers of prostate, large bowel and stomach. Among women, breast cancer is commonest, followed by cancers of large bowel, ovary and uterus overall. By design there were more women than men in the cohort so that relative cancer differentials cannot be assessed.

Table 3 shows mean daily intake and s.d. of the selected dietary variables by sex. The high consumption of vegetables, fruits and olive oil in the Greek population is evident, with olive oil also underlying the unusually high ratio of monounsaturated to saturated lipids in the diet. In this table the hazards ratios for cancer by the specified increments (approximately equal to the corresponding s.d.) of the indicated dietary variables are shown. The only significant associations with cancer overall are with the monounsaturated to saturated lipids ratio (inverse) and, unexpectedly, with intake of eggs (positive).

Table 4 shows cancer hazards ratios (95% confidence intervals) by MD score, overall and by sex. In comparison to those with poor MD adherence (score 0–3), those with intermediate (score 4–5) and high (score 6–9) adherence have progressively lower incidence of cancer overall. Excluding the first year of follow-up has essentially no effect on the estimates. In a continuous assessment, improved adherence by two points is associated with a highly significant (*P*-value=0.002) reduction in overall cancer incidence. The inverse association appears stronger among women, although the interaction by sex is not significant (*P*-value >0.50)

If residual confounding by smoking contributed to the inverse association (notwithstanding detailed control for smoking habits in the various models), one would expect it to be more evident for smoking-related cancers (Adami *et al*, 2008). However, using the same models for tobacco-related cancers, the hazard ratio for a 2-point increment in MD score was in fact less reduced than that for tobacco-unrelated cancers (see Table 4). Thus, residual confounding by aspects of smoking is unlikely to have played any major role in the inverse association found in this study.

## Discussion

In this sample of the general population of Greece, in the EPIC study, those who closely adhered to the traditional MD (operationalised through a simple 10-unit scale) had substantially and significantly lower incidence of overall cancer. The inverse association was exposure-dependent, it was almost identical after exclusion of the first year of follow-up and it was present among both women and men with no evidence for significant interaction by sex. The results indicate that closer conformity to the traditional MD by two units in the utilised scale (e.g. by substantially reducing meat intake and substantially increasing consumption of legumes *or* by substantially increasing intake of vegetables and substituting olive oil in the place of butter as an added lipid) is associated with a 12% reduction in the incidence of cancer overall.

Several reports have suggested that key MD components are associated with reduced cancer risk (Trichopoulou *et al*, 2000b; La Vecchia and Bosetti, 2006). Moreover, a few have used an MD adherence scale, conceptually similar to that used here. In an earlier study in the Greek EPIC cohort, with 97 deaths from cancer, a 2-point increase in an MD score as in the present study, was associated with a 24% reduction in overall cancer mortality with a wide 95% confidence interval of 2–41% reduction (Trichopoulou *et al*, 2003). Case–control studies in Italy using a slightly different score found that subjects with 6–8 MD characteristics compared to those with only 1–2, had more than 50% reduction in oropharyngeal, oesophageal and laryngeal cancer risks (Bosetti *et al*, 2003). In a cohort study in Sweden, among women aged 40–49 years old at enrolment who were followed-up for an average of 12 years, a 2-point increase in an MD score similar to that in the Greek studies, was associated with a borderline significant 16% reduction of overall cancer mortality (95% confidence interval −1 to 29% reduction); among women aged below 40 years at enrolment, no association was evident (Lagiou *et al*, 2006). In the US National Institutes of Health – American Association of Retired Persons Diet and Health study, with 5985 cancer deaths, cancer mortality among men and women with an MD score of 6–9 was significantly lower – by 17 and 12%, respectively – compared to those with a score from 0 to 3 (Mitrou *et al*, 2007). In that study, a 10-point alternate MD score created by Fung *et al* (2005) was used principally, but it was reported that the score used in the Greek studies (Trichopoulou *et al*, 2003) generated similar results. Thus, the observational studies converge in finding that adherence to an MD pattern is associated with lower cancer incidence.

This consistent evidence of a beneficial effect of the MD contrasts with the equivocal evidence for an important effect of individual foods, food groups, or nutrients on cancer risk as summarised in a recent review (World Cancer Research Fund/American Institute for Cancer Research, 2007). It is important to note that, in our study, MD is significantly and strongly inversely associated with cancer risk, whereas, the associations of the individual components of the MD with cancer are all nonsignificant, including the numerator and the denominator of the monounsaturated-to-saturated lipid ratio (as distinct from the ratio itself). Several explanations for this intriguing finding have been summarised (Jacques and Tucker, 2001;Trichopoulou *et al*, 2003): (1) individual components may have small effects that become evident only when they are integrated into a unidimensional score (2) there may be biological interactions between certain MD components and (3) for individual components, effects are examined against the background of average risk associated with other nutritional components, whereas a dietary score can evaluate the extremes of cumulative exposure (from 0 to 9) while minimising the ‘noise’ from individual components (Jacques and Tucker, 2001).

Strengths of this investigation are its prospective cohort design and its involving a general population sample in a Mediterranean country. The sample size and the duration of follow-up were adequate for studying the overall cancer occurrence. Moreover, a validated, interviewer-administered dietary questionnaire was used and cancer outcomes were all verified. The MD score employed has been consistently used in the Greek EPIC cohort (Trichopoulou *et al*, 2003, 2005b) and, with minor variations, in other large investigations in Europe (Trichopoulou *et al*, 2005a, 2007; Lagiou *et al*, 2006) thus providing indirect support to the robustness of the findings. Limitations include the facts that the sample size and duration of follow-up prevent examination of specific cancers, and that for 12% of the 851 cancer cases, date of death had to be substituted for diagnosis date. Finally, as in any observational study, concern remains about residual confounding, despite our controlling for smoking, level of education, physical activity and body mass index.

In conclusion, in a general population-based prospective cohort investigation, we have found evidence that adherence to the traditional MD is associated with markedly and significantly reduced incidence of overall cancer, which is appreciably larger than predicted from examining individual MD components.

## Figures and Tables

**Table 1:** Baseline characteristics of 25 623 cohort participants and 851 incident cancer cases, by sex and score in the Mediterranean diet scale: The Greek EPIC Study.

**Baseline characteristics**	**Men (*N*=10 582)**						**Women (*N*=15 041)**					
	**Score 0–3**		**Score 4–5**		**Score 6–9**		**Score 0–3**		**Score 4–5**		**Score 6–9**	
**(Number)**	**Cohort (3174)**	**Cases (142)**	**Cohort (4508)**	**Cases (180)**	**Cohort (2900)**	**Cases (99)**	**Cohort (5225)**	**Cases (174)**	**Cohort (6439)**	**Cases (165)**	**Cohort (3377)**	**Cases (91)**
*Mean (s.d.)*												
Age (years)	54.1 (13.7)	64.5 (9.8)	52.7 (12.7)	61.7 (9.9)	52.3 (11.9)	60.7 (10.2)	54.2 (13.2)	58.4 (11.1)	53.2 (12.3)	57.9 (11.8)	52.6 (11.5)	55.9 (11.4)
Formal education (years)	8.9 (4.8)	6.6 (3.9)	9.4 (4.8)	8.0 (4.9)	9.9 (4.8)	8.1 (4.7)	7.6 (4.7)	7.0 (4.7)	8.0 (4.7)	7.5 (4.6)	8.7 (5.0)	8.7 (4.9)
Height (cm)	169.4 (7.4)	167.4 (7.1)	169.9 (7.1)	168.8 (6.6)	170.4 (7.0)	169.1 (6.7)	155.8 (6.6)	155.4 (6.4)	156.3 (6.5)	156.1 (6.3)	156.9 (6.5)	156.8 (7.6)
Body Mass Index (kg m^−2^)	28.0 (3.9)	27.3 (3.9)	28.2 (3.8)	27.9 (3.9)	28.4 (3.7)	28.1 (4.1)	29.1 (5.3)	29.4 (5.5)	29.0 (5.2)	30.2 (5.6)	28.9 (5.1)	29.5 (5.7)
Physical activity (MET-hours per day)	34.5 (6.1)	32.7 (5.5)	35.4 (6.2)	33.7 (5.2)	36.0 (6.5)	34.5 (6.5)	34.8 (4.4)	34.8 (4.0)	35.6 (4.2)	35.1 (3.8)	36.2 (4.1)	36.4 (4.6)
												
*Percent*												
*Smoking status*												
Never	27	20	26	18	24	16	76	79	74	81	73	70
Former	32	35	35	40	37	46	7	7	8	5	9	11
Current	41	45	39	42	39	38	17	14	18	14	18	19
												
*Ethanol intake (g day*^−*1*^)												
<10	67	71	49	53	31	39	96	92	91	93	85	88
10 to <30	18	15	32	30	46	35	3	6	8	6	14	12
⩾30	14	14	19	17	23	25	1	2	1	1	1	0
												
*Supplement use*												
No	95	96	95	90	95	96	81	76	82	85	81	77
Yes	5	4	5	10	5	4	19	24	18	15	19	23

**Table 2:** Distribution of incident cancer cases, by major sites, among 25 623 men and women (188 042 person-years): The Greek EPIC Study.

**Cancer site**	ICD-O-2 codes*	**Men**	**Women**
Stomach	C16	35	13
Large bowel	C18-21	38	38
Liver	C22	19	9
Pancreas	C25	18	17
Lung	C34	116	19
Hematopoietic system	C42	25	18
Breast	C50	0	158
Cervix uteri	C53	—	21
Corpus uteri	C54	—	15
Ovary	C56	—	34
Prostate	C61	48	—
Kidney	C64	15	7
Bladder	C67	27	9
Brain	C71	11	14
Thyroid gland	C73	2	13
Lymphomas	C77	16	15
All other sites		50	30
Total		421	430

^*^World Health Organization (1990).

**Table 3:** Mean daily intake (±s.d.) of selected dietary variables and associated hazard ratios for incident cancer per indicated increments of intake with 95% confidence intervals (CI): The Greek EPIC Study.

**Intake (g day^−1^, except as indicated)**	**Men**	**Women**	**Increment^a^**	**Hazard ratio^b^**	**95% CI**
Vegetables	578.6±232.6	531.1±231.8	230	0.96	0.88–1.05
Legumes	10.2±7.3	7.8±5.7	6	0.98	0.92–1.05
Fruits – including nuts	383.2±214.5	375.6±206.7	205	1.01	0.93–1.09
Dairy products	221.2±147.2	214.7±146.0	140	1.03	0.96–1.10
Cereals	187.2±79.9	144.6±55.3	60	0.98	0.91–1.05
Meat	126.2±60.7	93.7±44.7	50	1.08	0.99–1.17
Fish	26.3±20.0	21.8±15.7	15	1.03	0.99–1.08
Ratio of monounsaturated to saturated lipids	1.8±0.5	1.8±0.5	0.5	0.91	0.85–0.98
Ethanol	18.0±23.9	3.3±6.2	10	1.00	0.96–1.04
Monounsaturated lipids	57.1±20.1	47.9±17.8	18	1.02	0.92–1.13
Saturated lipids	33.6±13.3	28.0±11.6	10	1.09	0.99–1.19
Polyunsaturated lipids	17.2±9.1	14.8±8.2	7	1.04	0.98–1.11
Olive oil	52.0±24.1	44.3±21.9	20	0.97	0.91–1.04
Potatoes	94.9±62.4	71.7±47.5	50	1.03	0.95–1.11
Eggs	18.3±13.1	15.3±10.2	10	1.07	1.01–1.13
Confectionery	25.3±19.8	22.4±17.5	15	1.01	0.95–1.08
Nonalcoholic beverages	376.0±243.3	285.8±200.6	220	0.99	0.91–1.08
Energy intake (kcal day^−1^)	2377±721	1898±580	650	1.05	0.98–1.14

a A round number close to the s.d. of the daily intake of the respective dietary variable.

b Stratified by sex. Adjusted for age (<35, 35–44, 45–54, 55–64, and ⩾65 years; categorically), years of schooling (⩽5, 6–11, 12, and ⩾13; categorically), smoking status (never smoker, former smoker, and five categories of current smoker: 1–10, 11–20, 21–30, 31–40, and ⩾41 cigarettes per day; ordered), body mass index (in ordered quintiles), height (in ordered quintiles), physical activity (MET-hours per day; in ordered quintiles), ethanol intake (per 10 grams; continuously), supplement use (no, yes) and total energy intake (in ordered quintiles) except for the model for energy intake.

**Table 4:** Hazard ratios^a^ for incident cancer (95% confidence intervals) by score in the Mediterranean Diet Scale among 25 623 cohort participants: The Greek EPIC Study.

	**Category of the mediterranean diet score**			
**Hazard ratios**	**Score 0–3**	**Score 4–5**	**Score 6–9**	**Per 2-point increment**
*For any cancer*	Reference	0.84 (0.72–0.98)	0.78 (0.64–0.94)	0.88 (0.80–0.95)
Smoking-related cancers^b^	Reference	0.83 (0.67–1.03)	0.86 (0.66–1.11)	0.91 (0.81–1.02)
Smoking-unrelated cancers^c^	Reference	0.86 (0.68–1.08)	0.70 (0.52–0.93)	0.84 (0.74–0.95)
				
Excluding first year of follow-up (all cancers)	Reference	0.85 (0.72–1.00)	0.76 (0.63–0.93)	0.88 (0.80–0.96)
				
*By sex (all cancers)*				
Men	Reference	0.96 (0.76–1.20)	0.83 (0.63–1.09)	0.91 (0.80–1.02)
Women	Reference	0.74 (0.59–0.92)	0.73 (0.56–0.96)	0.84 (0.74–0.95)

a Stratified by sex, where applicable. Adjusted for age (<35, 35–44, 45–54, 55–64, and ⩾65 years; categorically), years of schooling (⩽5, 6–11, 12, and ⩾13; categorically), smoking status (never smoker, former smoker, and five categories of current smoker: 1–10, 11–20, 21–30, 31–40, and ⩾41 cigarettes per day; ordered), body mass index (in ordered quintiles), height (in ordered quintiles), physical activity (MET-hours per day; in ordered quintiles), supplement use (no, yes), total energy intake (in ordered quintiles), as well as consumption of potatoes, eggs, confectionary, and non alcoholic beverages (per increment defined in Table 3).

b 454 cases of tobacco-related cancer sites (stomach, liver, pancreas, lung, hematopoietic system, cervix uteri, kidney, bladder, other sites).

c 397 cases for tobacco-unrelated cancer sites (large bowel, breast, corpus uteri, ovary, prostate, brain, thyroid gland, lymphomas).
